# Optimization and effects of different culture conditions on growth of *Halomicronema hongdechloris* – a filamentous cyanobacterium containing chlorophyll *f*

**DOI:** 10.3389/fpls.2014.00067

**Published:** 2014-02-25

**Authors:** Yaqiong Li, Yuankui Lin, Patrick C. Loughlin, Min Chen

**Affiliations:** School of Biological Sciences, University of SydneySydney, NSW, Australia

**Keywords:** cyanobacteria, chlorophyll *a*, chlorophyll *f*, *Halomicronema hongdechloris* (*H. hongdechloris*), light, photopigments

## Abstract

A chlorophyll *f* containing cyanobacterium, *Halomicronema hongdechloris *(*H. hongdechloris*) was isolated from a stromatolite cyanobacterial community. The extremely slow growth rate of *H. hongdechloris* has hindered research on this newly isolated cyanobacterium and the investigation of chlorophyll *f*-photosynthesis. Therefore, optimizing *H. hongdechloris* culture conditions has become an essential requirement for future research. This work investigated the effects of various culture conditions, essential nutrients and light environments to determine the optimal growth conditions for *H. hongdechloris *and the biosynthetic rate of chlorophyll *f*. Based on the total chlorophyll concentration, an optimal growth rate of 0.22 ± 0.02 day^-1^(doubling time: 3.1 ± 0.3 days) was observed when cells were grown under continuous illumination with far-red light with an intensity of 20 μE at 32°C in modified K + ES seawater (pH 8.0) with additional nitrogen and phosphor supplements. High performance liquid chromatography on *H. hongdechloris *pigments confirmed that chlorophyll *a* is the major chlorophyll and chlorophyll *f* constitutes ~10% of the total chlorophyll from cells grown under far-red light. Fluorescence confocal image analysis demonstrated changes of photosynthetic membranes and the distribution of photopigments in response to different light conditions. The total photosynthetic oxygen evolution yield per cell showed no changes under different light conditions, which confirms the involvement of chlorophyll *f *in oxygenic photosynthesis. The implications of the presence of chlorophyll *f* in *H. hongdechloris *and its relationship with the ambient light environment are discussed.

## INTRODUCTION

Cyanobacteria are oxygenic photosynthetic prokaryotes that convert CO_2_ into organic biomass by means of photosynthesis. Their metabolic flexibility to adapt and to thrive in various ecological niches is remarkable, and the optimal culture conditions of cyanobacteria are diverse among genera, species and strains (Pikuta et al., 2007; Singh, 2009). As with other bacteria, cyanobacteria have four different phases of growth: lag phase, exponential (or log) phase, stationary phase and the death phase (Fogg and Thake, 1987; Wood et al., 2005). The growth rate can be represented as the doubling time (the period of time required for the cell number or biomass to double). In addition to the direct measure of the growth rate of an organism by cell counting and/or the changes of total biomass (dry or wet weight), the growth rate of cyanobacteria can be measured indirectly using the changes of cellular components such as total organic carbon, lipids, protein, or chlorophyll (Hansmann, 1973; Moheimani et al., 2013). The total chlorophyll concentration has been widely used for the measurement of growth, particularly in the case of filamentous cyanobacteria, where the number of cells cannot be counted directly.

In order to maintain a culture successfully and to optimize the culture growth conditions, various environmental and nutritional parameters need to be taken into account. The most commonly studied parameters are light quality and quantity, pH, salinity, temperature, and macronutrients, mainly nitrogen (N) and phosphorus (P). Light is the major energy source for cyanobacteria and light quality and quantity strongly affect the light-harvesting systems and photosynthetic efficiency (Smith, 1986; Bibby et al., 2009; Chen and Scheer, 2013). *Synechocystis* sp. PCC6803 can be cultured easily under the laboratory conditions and is used as a model cyanobacterium for understanding the mechanism of photosynthesis. Recently, additional chlorophylls have been found in some cyanobacteria and the function of these novel chlorophylls in photosynthesis challenges the traditional belief of oxygenic photosynthesis: Chl *a* is the only chlorophyll that can involve the charge separation in the photosynthetic reaction centers (Miyashita et al., 1996; Chen et al., 2010). Cyanobacteria take advantage of having different compositions of photopigments to capture the available sunlight present in a particular ecological niche (Chen and Scheer, 2013; Hou et al., 2013). For example, *Acaryochloris marina* synthesizes chlorophyll *d* (Chl *d*) to capture far-red light that is the leftover light from phototrophs using Chl *a* existing in the upper stacked biolayers (Kühl et al., 2005; Loughlin et al., 2013). Chlorophyll *f* (Chl *f*) is the most recently identified chlorophyll, having the most red-shifted absorption peak of 707 nm found in oxygenic photosynthetic organisms to date (Chen et al., 2010). The presence of the red-shifted chlorophylls (Chl *d* and Chl *f*) enables oxygenic photosynthetic organisms to expand their absorbance spectral region beyond visible light region and to enhance their photosynthesis efficiency (Chen and Blankenship, 2011).

*Halomicronema hongdechloris *(*H. hongdechloris*) is the first reported filamentous cyanobacterium containing Chl *f* together with Chl *a* (Chen et al., 2012). A previous study suggested that phycobiliproteins are the main antenna system utilized by *H. hongdechloris* under white light conditions. However, Chl *f* is reported to be a “red-light-induced” chlorophyll with increased amount when *H. hongdechloris* is cultured under far-red light (Chen et al., 2012). *H. hongdechloris* offers an opportunity for exploring the function of Chl *f* in oxygenic photosynthesis. At the time when *H. hongdechloris* was isolated and purified, its doubling time was approximately 1 week (more than 150 h). Compared with the doubling time <20 h for *Synechocystis *sp. PCC 6803 (Saha et al., 2012), and ~33 h for *A. marina* (Miyashita et al., 2003) the growth rate of *H. hongdechloris* was extremely slow and became a hindrance to research on this newly isolated cyanobacterium and the investigation of Chl *f*-photosynthesis. With such slow growth, obtaining enough biomass of cells and Chl *f* is a major challenge for molecular, biochemical and biophysical studies. Therefore, optimizing *H. hongdechloris* culture conditions has become an essential requirement for future research.

Here we characterize *H. hongdechloris* growth kinetics and describe its light, pH, salinity, temperature and nitrogen/phosphorus requirements for the first time. The significantly improved growth rate of 0.22 ± 0.02 day^-1^ is achieved under enriched nitrogen and phosphorus nutrient conditions and continuous illumination of 730 nm monochromatic light with an intensity of 20 μE at 32°C. The influence of various light conditions and nutrient combinations on *H. hongdechloris* morphology and physiological features is observed using confocal fluorescence microscopy and transmission electron microscopy.

## MATERIALS AND METHODS

### CULTURE CONDITIONS

*Halomicronema hongdechloris* was isolated from stromatolites, Shark Bay, Western Australia (Chen et al., 2012). This strain was initially grown in modified K + ES seawater medium (**Table 1A**) with 100 rpm shaking under the control condition given in **Table 1B**. Seven days after inoculation, cells were homogenized using a glass homogenizer with K + ES seawater medium at a ratio of 1:10 (v/v) and used to inoculate 24 well plates (maximum capacity of 3.6 ml/well) with a final volume of 2 ml of cell culture per well. All cultures were inoculated with an equal cell amount containing a total chlorophyll concentration of 3.4 ± 0.4 μg/ml (*n* = 100).

**Table 1:** Control culture condition.

A. Modified K + ES seawater medium		
Major nutrient (mM)	NaNO_3_	2.35
	K_2_HPO_4_	0.03
Iron (μM)	Fe-Mn-EDTA	7.2
	Thiamine HCl	200
Vitamin (μg/L)	Biotin	1.5
	B12	1.5
Trace minerals (μg/L)	MnCl_2_.4H_2_O	178
	ZnSO_4_. 7H_2_O	2.3
	CoSO_4_. 7H_2_O	1.2
	Na_2_MO_4_.H_2_O	7.2
	CuSO_4_.5H_2_O	2.5
pH	TES^a^ (pH = 8.0)	25 mM
B. Culture condition		
Light quality		Far-red light^b^
Illumination time (h)		Continuous (24 h)
Light intensity (μE)		20
Temperature (°C)		32
Salinity (‰)		33^c^

a TES is 2-[[1,3-dihydroxy-2-(hydroxymethyl)propan-2-yl]amino]ethanesulfonic acid.

b Single wavelength light emitting diodes (LEDs) 730 nm with 20 nm half width and viewing angle 30° (Nanning Lvxing Light Electronics Co., Ltd., China).

c The sea salt concentration of 33.3 g in 1 L water is equivalent to 1 × natural seawater.

### GROWTH RATE, PIGMENT COMPOSITION AND STATISTICAL ANALYSIS

The growth rate of *H. hongdechloris* cultures was monitored using total chlorophyll concentration by sampling every 2–3 days over a period of 30 days. Cells from each well were sampled by centrifugation, and the methanolic total pigment extractions were directly used for spectral and high performance liquid chromatography (HPLC) analysis (Willows et al., 2013). All experiments were carried out on ice under dim green light to prevent photodamage of the pigment. Each experiment was repeated once in new culture plates and each sampling point had four technical replicates. Therefore, each point in the growth curve represents the mean of eight replicates.

Cell concentration was determined using total chlorophyll concentration as a proxy measure. The amount of total chlorophyll (Chl *a* and Chl *f*) was calculated according to the following equations based on the extinction coefficient published in Li et al. (2012).

(1) $$Chl\text{⁢}a\text{⁢}(µg/ml)=12.52A_{665-750nm}-2.28A_{707-750nm}$$

(2) $$Chl\,f(µg/ml)=12.78A_{707-750nm}-0.07A_{665-750nm}$$

The in vivo spectra of cells grown under different conditions were recorded using Shimadzu UV–Vis spectrophotometer (UV-2550) with a Taylor-sphere attachment (ISR-240A, Shimadzu, Japan). The cells were homogenized prior to measurement. The recorded spectra were smoothed using the Savitzky-Golay method with a window of <15 points (Origin version 8.0). Spectra were then re-plotted in Microsoft Office Excel 2007.

In this study, the growth rates (k) were the slope estimated based on the linear regression equation of the logarithm of total chlorophyll concentration in logarithmic growth phase in all conditions. The coefficient of determination, R^2^, determined using Microsoft Office Excel 2007 based on the linear regression equation. Cells grown under control culture conditions (**Table 1**) were used as the experimental reference for all factors tested in this study. When the relative growth rate is constant, the cell culture has a constant doubling time which can be calculated based on:

(3) Doubling⁢time(t)=ln⁢2/k

where k (day^-1^) is the growth rate.

### STATISTICAL ANALYSES

The mean values, confidence intervals and SD values of the replicates for each treatment were calculated by Microsoft Office Excel 2007. The SE of growth rate generated by linear regression were calculated as described in Kenney and Keeping (1962). The effects on growth characters as growth rate and cell sizes caused by different growth conditions were analyzed by ANOVA using R (version 3.0.0). The post hoc multiple comparisons were made by a F-test if a significant difference was detected. The level of significance was set at 0.05 for all testing conditions. All errors quoted in this study are ±95% confidence intervals with the numbers of replicate presented in brackets (n).

### VARIATIONS OF LIGHT CONDITIONS

The effects of light qualities and quantities for H. hongdechloris were examined by setting seven different light conditions at various intensities: far-red light (FR, 730 nm LEDs with 20 nm half width and 30° viewing angle) with intensities ranging from 10 to 60 μE; red (RL, 650 nm LEDs with 20 nm half width at a 15° viewing angle), orange-red (OR, 625 nm LEDs with 20 nm half width at a 15° viewing angle), green (520 nm) LEDs with 20 nm half width at a 30° viewing angle), or blue (470 nm) LEDs with 20 nm half width at a 30° viewing angle) light with intensities of 10 and 20 μE; white light (cool white fluorescent light, WL) with intensities of 10–100 μE. LEDs lights utilized in this study are all mono-wavelength LED clusters (Nanning Lvxing Light Electronics Co., Ltd., China). The radiation spectra of various light conditions were monitored using an USB2000+ Miniature Fiber Optic Spectrometer (Ocean Optics, Inc., Australia). Different light intensities were obtained by adjusting the distance between sample and light source, and monitored using a SKP200 light meter (Skye Instruments Ltd., UK) for the OR, RL and FR lights or a Quantum LI-light meter (LI-COR, Inc., USA) for the other light sources. All illumination was continuous. Except for differences in light conditions, cells were all incubated in the same conditions as the control (**Table 1**).

### RELATIONSHIP BETWEEN TOTAL CHLOROPHYLL AND WET WEIGHT OF CELLS

The wet weight of cells grown under FR light/10–20 μE or WL light/40–60 μE at the period of exponential phase was obtained by rinsing the cells using milliQ water and removing any remaining surface water by vacuum filtration before weighing. Subsequently, total chlorophyll was extracted from the same samples using 100% methanol (HPLC grade) and the total chlorophyll concentration was determined as described above (Eqs 1 and 2). The rinsed cells were extracted by 100% methanol several times until no detectable pigment was observed in the absorption spectrum (absorbance reading at 665 nm < 0.05). This process was repeated (*n* = 10) for each culture treatment using different amount of cells ranging from 0.25 to 1.50 g. The relationship between total chlorophyll and wet weight were re-plotted in Microsoft Office Excel 2007.

### VARIATIONS OF SELECTED ENVIRONMENTAL AND NUTRITIONAL FACTORS

The environmental factors tested in this study were: pH, temperature and salinity. The pH of the medium was maintained using Good’s buffers (Good et al., 1966) at a concentration of 25 mM: 2-(N-morpholino)ethanesulfonic acid (MES; pH 6.0), TES (pH 7.0 and 8.0), and N-Cyclohexyl-2-aminoethanesulfonic acid (CHES; pH 9.0 and 10.0). To determine the best culture temperature, cells were cultivated under four selected temperatures (20°C, 27°C, 32°C, and 39°C). The effects of salinity on the growth of the cells were characterized by monitoring the growth rates in modified K + ES medium with different concentrations of commercial premium Sea Salt (AQUASONIC, Australia) from 0.0 g (fresh water) to 3.3 g per 100 ml medium (1 × salinity of nature seawater = 33‰). When the salt concentration of the medium exceeded 1 × seawater, additional NaCl was used to reach the required level of salinity. In all cases, apart from the parameter being tested, conditions were the same as the control condition (**Table 1**).

The effect of different inorganic nitrogen sources ((NH_4_)_2_SO_4_, NH_4_Cl, NaNO_3_, NaNO_2_) and ratio of N:P (mM:mM) on the growth of *H. hongdechloris *were also examined. The ratio of N:P was varied from 7.8 to 780 (with control conditions from **Table 1** being 78) by maintaining the control nitrogen concentration and increasing the phosphorus concentration by 5- or 10-fold or maintaining the control phosphorus concentration and increasing the nitrogen.

### PHOTOSYNTHETIC OXYGEN EVOLUTION RATE

The oxygen evolution rates of *H. hongdechloris *cultures were measured using a Hanstech DW2/2 Liquid-Phase Oxygen Electrode Chamber with a circulating thermostat-regulated water bath. All oxygen evolution rate experiments were done at 25°C, except the high temperature measurement (32°C). To avoid large filamentous cell aggregation, the cell culture was homogenized and re-cultured under the same conditions for approximately a week until their biological activity recovered and cell growth was in the exponential phase. One ml of pre-homogenized cell culture with a total chlorophyll content of 4–8 μg was added to the chamber for oxygen evolution rate measurement. The light source was provided by either a Leica Pradovit color 250 (Autofocus) projector with different transmission filters or a FR light (730 nm with 20 nm half width and 30° viewing angle, Nanning Lvxing Light Electronics Co., Ltd., China).

### CONFOCAL MICROSCOPY

The cell morphology of exponential phase *H. hongdechloris* cells grown under different light regimes was examined on a Zeiss LSM Pascal 410 confocal microscope (Oberkochen, Germany) using an Achroplan 63 × water immersion objective or Plan-Neofluar 100 × oil immersion object. Cells were excited using a 458 nm argon laser and autofluorescence was collected using either a 600–680 nm band-pass filter or a 692 nm long-pass filter.

### TRANSMISSION ELECTRON MICROSCOPY

Exponential phase cells grown under FR and WL conditions were fixed in 2.5% (v/v) glutaraldehyde with 6% (w/v) sucrose in 0.1 M phosphate buffer pH 7.5 overnight at 4°C. Cells were rinsed 3 × 10 min in the same buffer, and then embedded in 1% (w/v) low melting agarose and further fixed in 1% (w/v) osmium tetroxide with 6% (w/v) sucrose in 0.1 M phosphate buffer pH 7.5 overnight at room temperature. Cells were rinsed 3 × 10 min with milliQ water, and then dehydrated in a series of ethanol concentrations from 50 to 100% for 10 min each. The dehydrated cells were infiltrated with Spurr’s resin (Proscitech, Australia) and embedded in gelatin capsules. The samples were polymerized at 65°C for 24 h. Semi-thin sections (1 μm) were cut using a glass knife on an ultramicrotome (Ultracut T, Leica, Austria), mounted and dried on glass slides and stained with 0.5% (w/v) Toluidine blue. Ultrathin sections (70 nm) were cut on the ultramictrotome, placed on 200 mesh thin bar copper grids (Proscitech, Australia) and stained with 2% uranyl acetate for 10 min and lead citrate (Reynold’s) for 10 min. All sections were visualized using a transmission electron microscope (JEOL 1400, JEOL Ltd.) at 120 kV. Images were captured using a Gatan Erlangshen ES500W camera and processed using Gatan DigitalMicrograph^TM^ software (Gatan, USA).

## RESULTS

**FIGURE 1:**
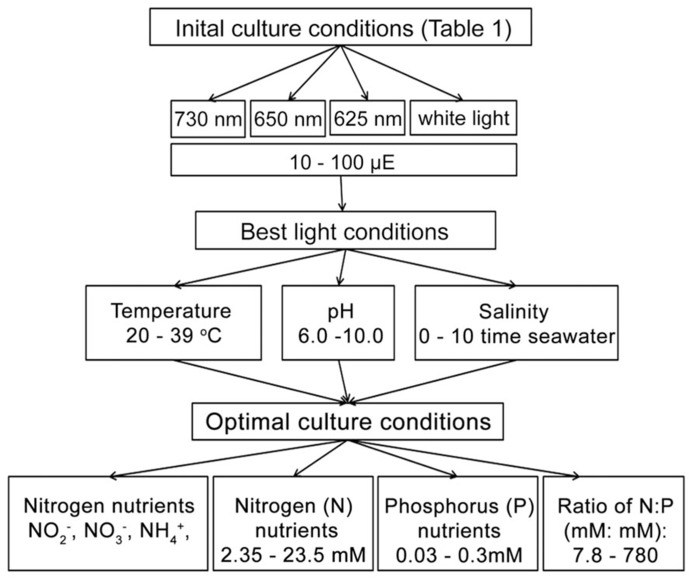
**Experimental scheme**.

To determine the optimal culture conditions, more than 20 different light conditions, covering four different light sources with a series light intensities were tested initially to investigate the best light condition for growing *H. hongdechloris *under laboratory conditions (**Figure 1**). The optimized light conditions were applied in the subsequent studies on optimizing the growth factors. Optimization of environmental and nutritional conditions was performed in a step-wise fashion as follows: optimal culture salinity was determined following by pH, then temperature and finally nitrogen and phosphorus requirements were examined under these optimized conditions (**Figure 1**).

### THE LINEAR RELATIONSHIP BETWEEN TOTAL CHLOROPHYLL AND BIOMASS

**FIGURE 2:**
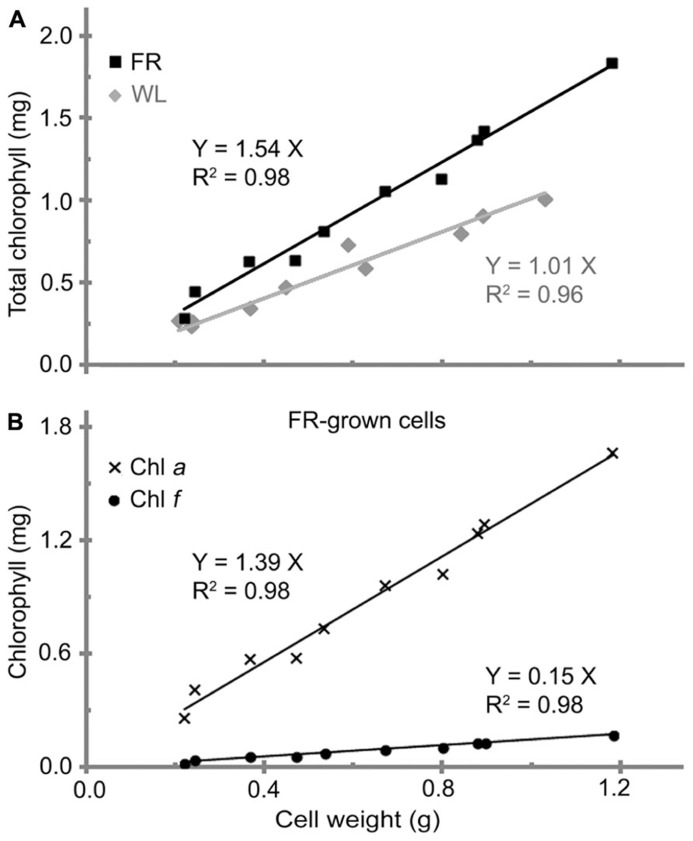
**The relationship between cell weight and chlorophyll contents in *H. hongdechloris*.** **(A)** Comparison of total chlorophyll per cell weight (mg/g) for white light-grown (WL, gray line) and far-red light-grown (FR, black line) cells. **(B)** Comparison of Chl *a* (line with crosses) and Chl *f* (line with dots) per cell wet weight (mg/g) for FR-grown cells.

A linear relationship between the total chlorophyll and cell wet weight for *H. hongdechloris* cells grown was observed under both FR light/10–20 μE and WL/40–60 μE conditions (**Figure 2A**). The significant linear relationship indicates that the content of total chlorophyll is relatively constant per cell under the same light conditions. It is of interest to note that the chlorophyll content per cell was ~1.54 (mg chlorophyll) g^-1^ cell wet weight for the cells grown under FR light, which is about 50% more chlorophyll content compared to the cells grown under WL light. The cells grown under WL light have about 1.0 mg chlorophyll/g cell wet weight (**Figure 2A**). Both Chl *a* and Chl *f* showed a linear relationship with the cell wet weight, 1.39 (mg Chl *a*) g^-1^ and 0.15 (mg Chl *f*) g^-1^ cell wet weight in FR light-grown cells (**Figure 2B**). Cell size did not vary under the different light qualities according to the microscopic observation based on the average measurement of >100 cells per treatment (**Table 2**). There are no significant differences observed in both cell length (*F*(3,346) = 2.17, *p* = 0.091 > 0.05) and width (*F*(3,346) = 0.93, *p* = 0.428 > 0.05) according to a one-way ANOVA analysis (**Table 2**). Hence, the total chlorophyll content is used as a measure of growth throughout this report.

**Table 2:** Cell sizes of *H. hongdechloris* cells grown under different light conditions.

		Light conditions			
		White (WL)	Far red (FR)	Orange red (OR)	Red light (RL)
Cells Size	Length	1.81 ± 0.20 (*n* = 100)	1.86 ± 0.23 (*n* = 100)	1.80 ± 0.13 (*n* = 100)	1.79 ± 0.25 (*n* = 50)
	Width	0.86 ± 0.12 (*n* = 100)	0.88 ± 0.06 (*n* = 100)	0.88 ± 0.07 (*n* = 100)	0.87 ± 0.08 (*n* = 50)

### THE INFLUENCE OF VARIOUS LIGHT CONDITIONS ON CELL GROWTH

**FIGURE 3:**
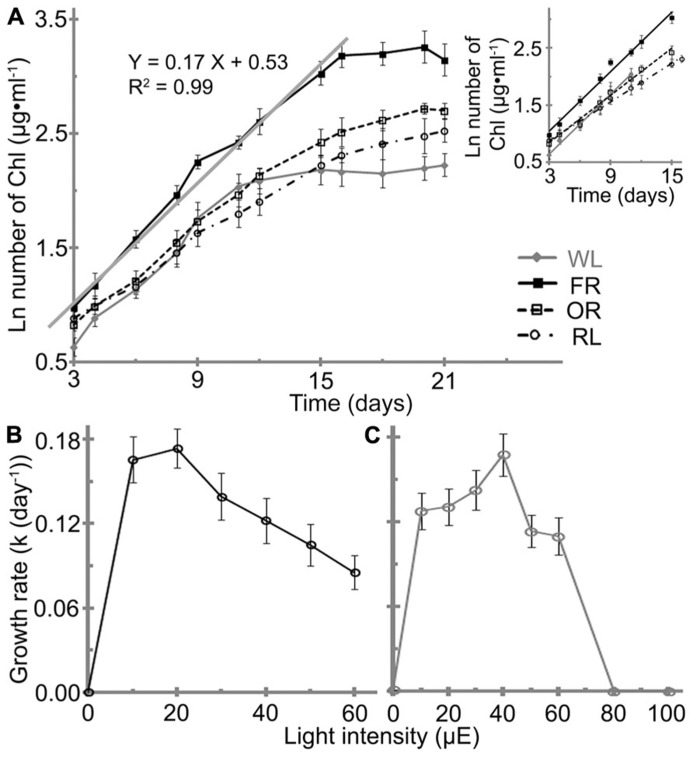
**Effects of light conditions on the growth profile of *H. hongdechloris*.** **(A)** Growth profile of *H. hongdechloris* under different light conditions. Optimal intensities for all red/orange LED lights were 20 μE, but 40 μE for white light. The solid gray line and equation denotes the trend line of linear regressions of the logarithm of total chlorophyll concentration in the exponential phase under the FR/20 μE condition. **A** insert: Comparison of the trend lines of linear regressions of the logarithm of total chlorophyll concentration in the exponential phase for cells grown under different light conditions. FR, far-red; OR, orange-red; RL, red light and WL, white light. Error bars represent SDs (*n* = 8). Effects of **(B)** FR and **(C)** WL light intensities on the growth rate of *H. hongdechloris*. Error bars represent SE (*n* = 8).

*H. hongdechloris* pigment composition has previously been reported to change in response to two light conditions: WL light/20 μE and 720 nm LED light/10–15 μE (Chen et al., 2012). *H. hongdechloris *cells grow under the all tested orange to red LED lights and also WL light (**Figure 3A**). However, no growth was observed when cells were grown under green or blue light at intensities of either 10 or 20 μE (data not shown). The growth rates under the white and red light conditions examined vary considerably with an increased growth rate observed in the following order: FR/20 μE ≥ WL/40 μE > OR/20 μE > RL/20 μE based on total chlorophyll content.

The highest growth rate (*k*) of ~0.17 ± 0.01 day^-1^ was obtained under either FR/20 μE or WL/40 μE (**Figures 3B,C**). However, the shape of growth curve and the maximum chlorophyll content at stationary phase are different under the two light conditions. Under WL/40 μE, the *H. hongdechloris* culture reaches stationary phase at ~11 days after a relatively short exponential phase of ~7 days, resulting in a significantly lower biomass (based on total chlorophyll content) compared with cultures grown under red light conditions. Under FR/20 μE and other red light conditions, the exponential growth phase was extended to >15 days and the maximum chlorophyll content of ~26 mg ml^-1^was achieved (**Figure 3A**). Surprisingly, the growth rate decreased sharply when the light intensity was increased above 30 μE for FR light and 50 μE for WL light (**Figures 3B,C**). The lowest growth rate of ~0.08 ± 0.01 day^-1^ was observed when the FR light intensity was increased to 60 μE, the highest light intensity using the available LED light source in the laboratory. No cell growth was detected when WL light intensity was increased to 80 μE or greater (**Figures 3B,C**). These data suggest *H. hongdechloris* prefers a low light intensity and to use FR light for growth.

### THE INFLUENCE OF VARIOUS CULTURE CONDITIONS ON CELL GROWTH

**FIGURE 4:**
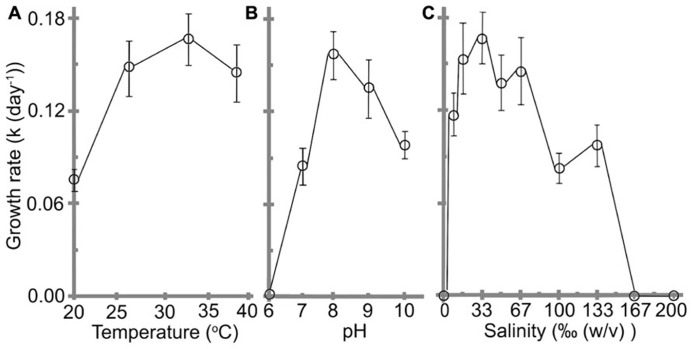
**Effects of (A) temperature, (B) pH, and (C) salinity on the growth rate of *H. hongdechloris*.** Error bars represent SE (*n* = 8).

Understanding the impacts of environmental as well as other physico-chemical parameters are important for a newly purified cyanobacterial culture. Effects of pH, temperature, and salinity on the cell growth are shown in **Figure 4**. The cells grew at temperature ranges from 20 to 39°C with a best *k* of 0.17 ± 0.02 day^-1^and *t* of 4.2 ± 0.4 days were obtained at 32°C, which was assigned as the optimal temperature (**Figure 4A**). No significant reductions were observed when the temperature was reduced to 27°C or increased to 39°C (*F*(2,21) = 1.07, *p* = 0.3617).

The optimal pH for *H. hongdechloris* culture was determined to be pH 8 (*k* = 0.16 ± 0.02 day^-1^, *t* = 4.4 ± 0.4 days; **Figure 4B**). Cells died in acidic (pH ≤ 6.0) media conditions (**Figure 4B**); however, they were tolerant of alkaline media conditions up to pH 10, the highest pH tested in this study, although the growth rate is much lower than the cells under optimal pH conditions (**Figure 4B**). These data highlight the relative acid sensitivity of *H. hongdechloris*.

Variations in salinity impact a cell’s ability to absorb water and nutrients from the medium (Moisander et al., 2002). Culturing of *H. hongdechloris* in different salinities revealed the best *k* of 0.17 ± 0.02 day^-1^ (*t* = 4.2 ± 0.4 day) is obtained in 1× salinity of natural seawater (33‰(w/v) artificial sea salt); however, similar growth rate was observed in seawater salt concentration between 25‰(w/v) and 67‰(w/v); (**Figure 4C**). *H. hongdechloris *exhibited resistance to the changes in salinity ranging from 8 to 133‰(w/v); however, it cannot survive in the K + ES fresh water medium or salinity higher than 167‰(5 × the salinity of natural seawater; **Figure 4C**).

### THE INFLUENCE OF VARIOUS NUTRIENT CONDITIONS ON CELL GROWTH

**FIGURE 5:**
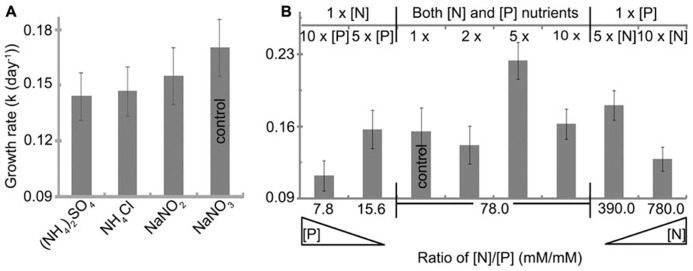
**Effect of (A) nitrogen source and (B) nitrogen and phosphorus concentration on the growth rate of *H. hongdechloris*.** All concentrations are relative to the concentrations outlined in **Table 1**. Error bars represent SE (*n* = 8).

All nitrogen sources studied were initially provided to *H. hongdechloris* at 2.35 mM nitrogen content (**Table 1**). The best growth rate of 0.17 ± 0.01 day^-1^(*t* = 4.1 ± 0.4 day) was observed using nitrate (NaNO_3_) as a nitrogen source, although there were no significant difference among the nitrogen nutrient forms, NH_4_^+^, NO_2_, and NO_3_ (**Figure 5A**). NaNO_3_was used as the best nitrogen nutrient source for *H. hongdechloris* culture.

The amounts and ratio of nitrogen and phosphorus are an important correlated requirement for algal culture (Smith, 1983; Stockner and Shortreed, 1988; Dolman et al., 2012). **Figure 5B** illustrates that growth rates were affected by changing the ratios of N:P (mM:mM)) and the total concentration of nitrogen and phosphorus nutrients. The highest growth rate of 0.22 ± 0.02 day^-1^ and a doubling time of 3.1 ± 0.2 days were observed when both total nitrogen and phosphorus nutrient concentration were increased fivefold compared with the standard amount in K + ES but the ratio of N:P remained the same, i.e., the best nutrient condition is: 11.75 mM nitrogen in the form of NaNO_3_, 0.15 mM phosphorus in the form of PO_4_^3-^ and the ratio of N:P is 78 (**Figure 5B**). Under these conditions, the maximum biomass, calculated based on the rate of total chlorophyll to cell wet weight shown in **Figure 2**, was >35.7 mg ml^-1^ (data not shown), which is almost double the biomass observed in cells grown under normal N:P concentrations (**Table 1**). The imbalance of the ratio of N:P, either <7.8 or >780 inhibited the growth of *H. hongdechloris* (**Figure 5B**).

### CHARACTERIZED PHYSIOLOGICAL PROPERTIES

#### Photopigment composition

**FIGURE 6:**
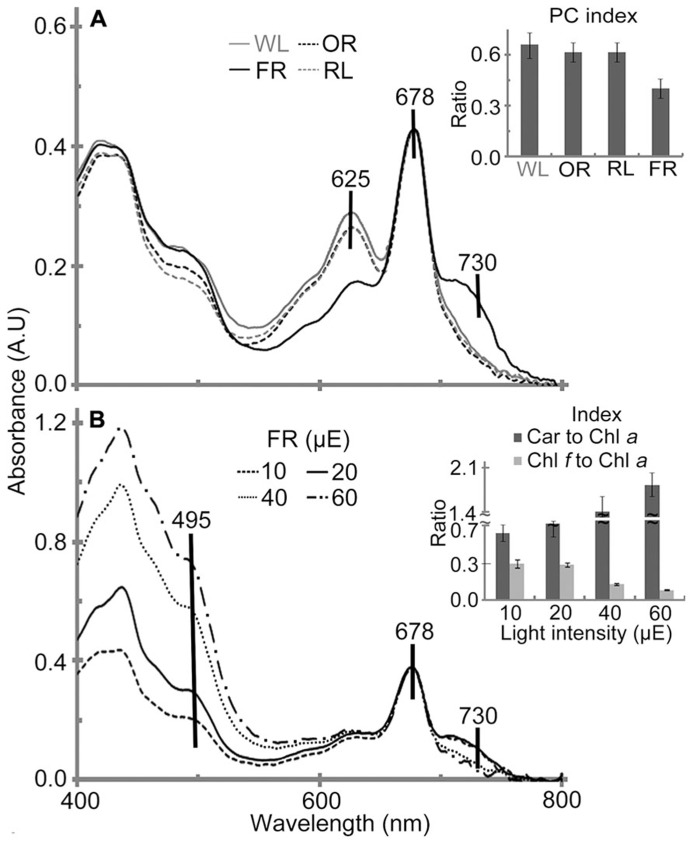
***In vivo* absorption spectra of *H. hongdechloris *grown under different light qualities (A) and different intensities of FR light (B).** Absorption peaks corresponding to carotenoid (495 nm), phycobiliprotein (625 nm), Chl *a *(678 nm) and Chl *f *(730 nm) are indicated. **A** insert: PC index [phycobiliprotein to Chl *a *ratio (*A*_625-780 nm_/*A*_678-780 nm_)] of *H. hongdechloris *grown under different light qualities; FR, far-red; OR, orange-red; RL, red light and WL, white light. **B** insert: Car to Chl *a *(carotenoid to Chl *a*, *A*_495-780 nm_/*A*_678-780 nm_) and Chl *f *to Chl *a *index (*A*_730-780 nm_/*A*_678-780 nm_) index of *H. hongdechloris *under different intensities of 730 nm light. All data represent the means of five replicates (*n* = 5).

*In vivo *absorption spectra of *H. hongdechloris *cells indicated that Chl *a* (678 nm peak) and phycobiliprotein (625 nm peak) are the main photopigments in cultures grown under WL, OR or RL conditions (**Figure 6**). The absorption spectral profiles are similar to most other typical cyanobacteria using Chl *a* and phycobiliproteins. Further HPLC analysis confirmed that no detectable Chl *f* existed in cultures grown under WL, OR or RL conditions (data not shown). However, the pigment composition of *H. hongdechloris* under FR light is unique, having an absorption spectrum indicative of the presence of Chl *a* (678 nm) and Chl *f* (730 nm), with a limited amount of phycobiliproteins (**Figure 6A**), which is consistent with the previous report (Chen et al., 2012). Despite the presence of Chl *f* in FR light-grown cells, Chl *a* still remains the main chlorophyll of *H. hongdechloris *under all light conditions tested (**Figure 6A**).

**FIGURE 7:**
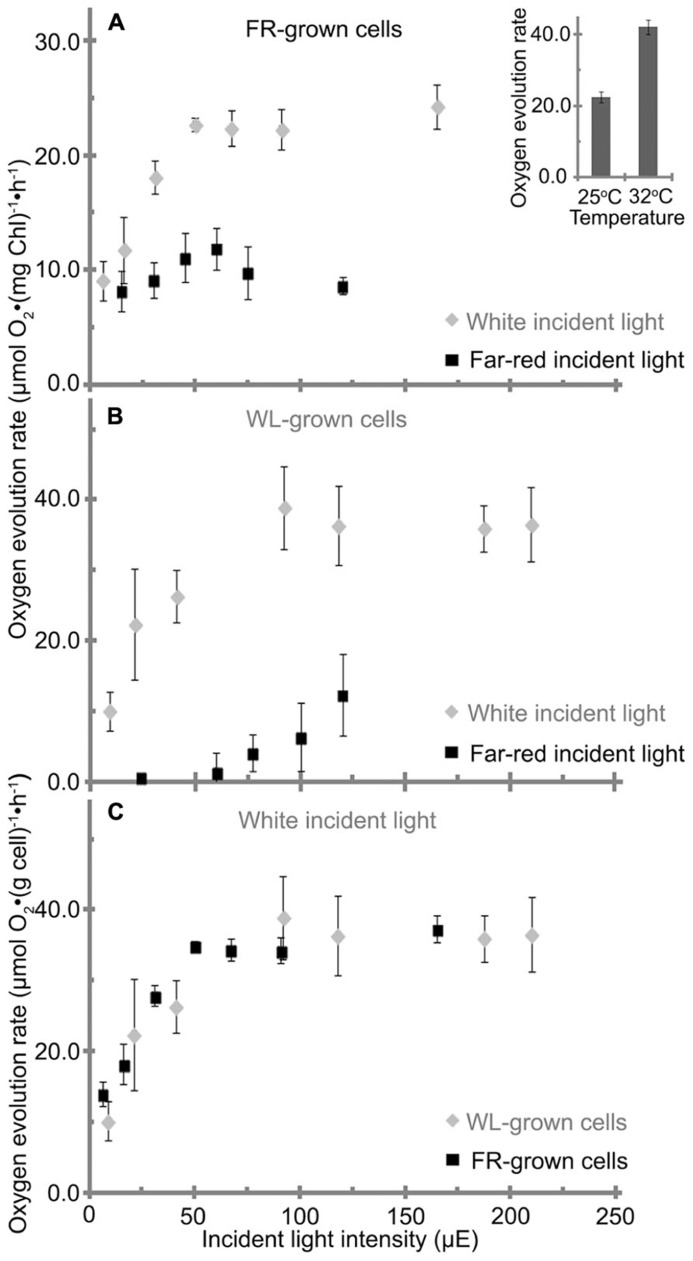
**Oxygen evolution rate of *H. hongdechloris *grown under (A) far-red light (FR) or (B) white light (WL), with either FR or WL light as the incident light source as indicated.** Data are based on total chlorophyll content. **A** insert: oxygen evolution rate of *H. hongdechloris *cells at 25°C or 32°C with 67 μE white light illumination. **(C)** The oxygen evolution rate of *H. hongdechloris *cells grown under either FR or WL conditions based on biomass, using white light as the incident light source. Error bars represent SDs (*n* = 9).

To enhance the content of Chl *f* and the total biomass, different light intensities of FR light were tested and compared with the equivalent intensities of white light. The highest Chl *f*/Chl *a *index (*A*_730-780 nm_/*A*_678-780 nm_) of 0.29 is observed under the FR light intensities of 10–20 μE (**Figure 6B**). Increased FR light intensities resulted in a decreased Chl *f*/Chl *a *index of 0.13 and the increased carotenoids to Chl *a* index (Car/Chl *a*, *A*_495-780 nm_/*A*_678-780 nm_), which may indicate the culture was under high-light stress. Interestingly, the cells grown under WL light showed a more stable pigment content beside the increased Car/Chl *a* index under increased light intensities. The highest Car/Chl *a* index of ~1.9 was observed in the cells grown under FR light/60 μE light, suggesting the high-light stressed situation for *H. hongdechloris* cells (**Figure 7B** insert). The relatively stable amount of phycobiliprotein and the dramatic changes in Chl *f* concentration (**Figure 6B** insert) observed in cells grown under different intensities of FR light suggest that Chl *f* plays an important role in the capture and use of FR light for driving photosynthetic reactions. Increases in the Car/Chl *a* index observed in the cells under possible high-light stress agree well with the decreased growth rate for the cells grown under these higher intensities of either FR or WL light (**Figures 3B,C**). The optimized light condition for *H. hongdechloris* culture is FR light with lower light intensity of 10–20 μE.

#### Photosynthetic activities (photosynthetic oxygen evolution rate)

*H. hongdechloris* cells grown under FR light and WL light conditions were subjected to oxygen evolution measurements. When WL was used as the incident light source, the FR light-grown cells had a maximum oxygen evolution rate of 23 μmol O_2_ (mg Chl)^-1^ h^-1^ and reached its light saturation point at ~50 μE at 25°C (**Figure 7A**). However, under the same conditions the WL light-grown cells had a higher maximum oxygen evolution rate of 39 μmol O_2_ (mg Chl)^-1^ h^-1^ and reached its light saturation point at about 90 μE at 25°C (**Figure 7B**). *H. hongdechloris *cells cultured in both conditions show lower oxygen evolution rates than typical cyanobacteria (Lee et al., 1998; Gloag et al., 2007; Milligan et al., 2007). Given the slower growth rate of *H. hongdechloris*, this lower oxygen evolution rate is unsurprising.

When a FR light was used as the incident light source for detecting oxygen evolution, the maximum evolution rate for FR light grown cells is only about 12 μmol O_2_ (mg Chl)^-1^ h^-1^, ~50% oxygen evolution rate using WL as incident light at the equivalent intensity (**Figure 7A**). White light grown cells did not show any detectable oxygen production under illumination of FR light when the light intensity was <60 μE (**Figure 7B**). This result suggests that Chl *f* in *H. hongdechloris *plays an important role in capturing FR light and transferring the energy to the reaction center for photosynthetic activity. As reported in **Figure 2**, the chlorophyll content in WL light-grown cells is lower than that in FR light grown cells (**Figure 2A**). Taking this parameter into account, a similar maximum oxygen evolution rate of about 35–40 μmol O_2_ (cell weight g)^-1^ h^-1^ is observed for cells grown under both light conditions (**Figure 7C**) when using WL as the incident light. The oxygen evolution rate is almost doubled when the measurement temperature is increased from a standard 25 to 32°C (**Figure 7A** insert), which is in agreement with the optimized *H. hongdechloris* culture temperature (**Figure 7A**).

#### The morphology of H. hongdechloris grown under various light conditions

**FIGURE 8:**
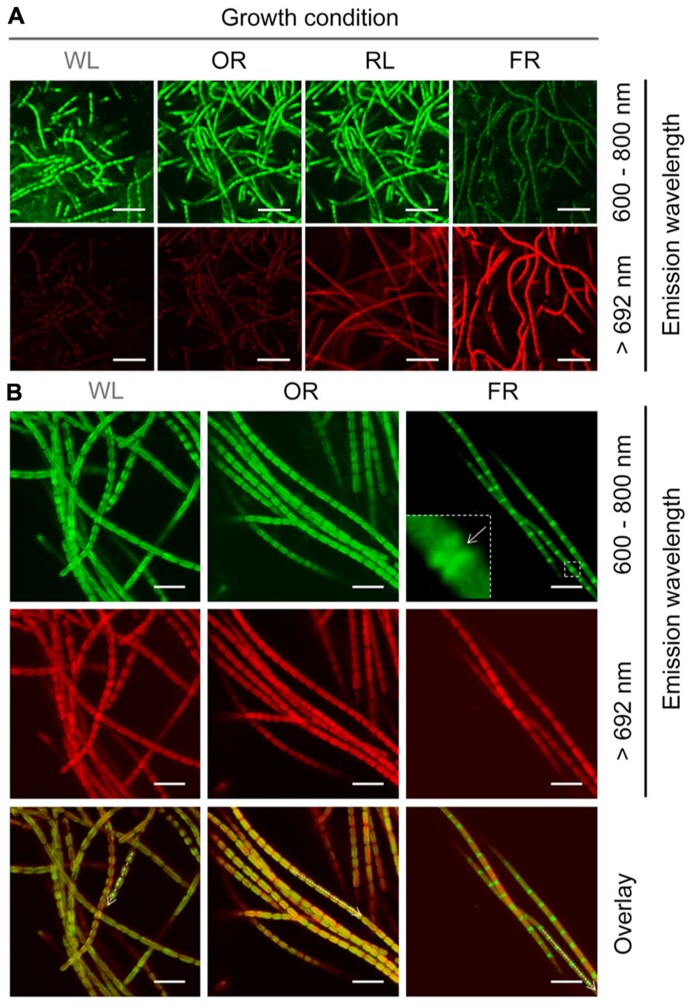
**Confocal fluorescence images of *H. hongdechloris *cells grown under different light conditions.** Autoflourescence of cells excited with 458 nm light were collected using either a 600–680 nm band-pass filter or a 692 nm long-pass filter (>692 nm). **(A)** Confocal fluorescence images of cells grown under WL, FR, OR, or RL light. All data were collected using identical laser intensity and gain settings. Bar = 10 μm. **(B)** Confocal fluorescence images of cells grown in WL, OR, or FR light conditions. Fluorescence emission at 600–680 nm was detected uniformly around the periphery of cells grown in other selected lights whereas in FR light grown cells this fluorescence was concentrated in the septa. Intensities of the different fluorescence wavelengths along the axis of the dotted arrows in the overlay are plotted in the lower panel. Different laser intensities and gain settings were used in order to collect normalized fluorescence intensities. Bar = 5 μm.

Different wavelength pass filters were applied for detecting the fluorescence generated from different photopigments using confocal fluorescence microscope. The fluorescence detected by an emission band pass filter of 600–680 nm is mainly generated from phycobiliproteins (Blankenship, 2002; Collins et al., 2012), and the fluorescence detected using a 692 nm long pass filter is largely due to the presence of Chl *f*. Strong >692 nm fluorescence was only observed in *H. hongdechloris *cells grown under FR light, and not in the cells grown under WL, OR or RL light (**Figure 8A**). This >692 nm fluorescence observed from FR light-grown cells was distributed evenly around the peripheral thylakoid membranes (Th) of the cells, suggesting that Chl *f-*binding protein complexes are also distributed through these membranes (**Figure 8B**). Using the emission band pass filter (600–680 nm), strong and uniform fluorescence was observed in cells grown under white, orange-red or red light. However, the fluorescence recorded using this filter was much weaker for the cells grown under FR light; and strikingly, the strongest fluorescence was localized at the septa region. This phenomenon was also observed in our previous report (Chen et al., 2012). Both 600–680 nm and >692 nm fluorescence patterns for white and OR light grown cells overlayed well with one another; however, for FR light grown cells, a clear inverse pattern of fluorescence was observed (**Figure 8B**).

**FIGURE 9:**
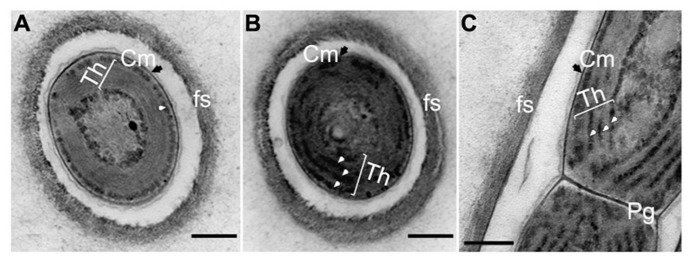
**Transmission electron micrograph of ultrathin section of *H. hongdechloris *cells grown under white and far-red light conditions.** **(A)** Cross section of a cell grown under FR light; **(B)** Cross section of a cell grown under white light; **(C)** Longitudinal section of the intersection (septum region) between filamentous cells grown under white light. White arrows indicate phycobilisome-like structures filling the stromal side of the thylakoid membranes (Th). Pg, peptidoglycan layer; fs, fibrils sheath and Cm (black arrows), cytoplasmic membrane. Bar = 0.2 m.

Comparison of the ultrastructure of *H. hongdechloris *grown under either WL or FR light demonstrated no obvious difference in cell morphology, apart from a slight difference in the thicknesses of appressed Th (**Figure 9**). The cells grown under WL demonstrate that phycobilisome-like structures are uniformly distributed along the Th (white arrows in **Figures 9B,C**). The Th in the FR light grown cells were more appressed, which seems likely consistent with the reduction in the amount of phycobilisomes between the Th (**Figure 9A**). Conversely, the phycobilosome-like structures can be only observed between the plasma membrane and Th in the cells grown under FR light (**Figure 9A**). Under both FR and WL light conditions, cells have a filamentous structure surrounded by a fibrous sheath (fs)-like layer with a thickness of 0.08–0.15 μm (*n* = 200) and a peptidoglycan layer (Pg) shared between the cells. No branching was observed (**Figure 9**).

## DISCUSSION

In liquid culture, *H. hongdechloris* has a propensity to attach to surfaces and to aggregate. This strong adhesion creates difficulties for cell counting and other measurements. Cells used in this study were homogenized before inoculation in order to obtain an equal number of cells at the start of each experiment (total chlorophyll concentration of 3.4 ± 0.4 μg/ml). A consequence of this homogenization process is some apparent damage to the cells that contributes to the lag phase after inoculation (**Figure 3A**). To avoid the effects of this reduction, growth rate was only calculated using the data collected from exponential phase, where more than six measuring points are recorded. All growth curves presented in this study start from day three after lag phase. The coefficient of determination, *R*^2^, was over 0.95 in most of the conditions, which represents over 95% of data that is closest to the line of best fit. The statistical analysis indicates that the calculation of growth rate is reliable. Furthermore, the reproducibility of the results is confirmed by the consistent doubling time of the control treatment, which was always in the range of 4.1–4.4 days (*n* = 10) throughout the whole study.

All growth curves are generated from at least two biological repeats and four technical repeats, and each set of measurement requires minimum five weeks for completion. Such time-consuming experiments forced us to design one-way optimizing strategy, that is, we tested one environmental element at once and used the optimized condition for the next test (**Figure 1**). Comparison of the maximum biomass based on the total chlorophyll under a particular test condition may not be an appropriate method since we observed a difference in the total chlorophyll present in cells grown under FR light compared to WL light (**Figure 2**). However, the measured growth rate should be reliable as there is a linear relationship observed between total chlorophyll content and biomass under the same light conditions, and therefore chlorophyll content can be used as a proxy measure for biomass for each light condition. Additionally, there were no observable differences in the cell size or filamentous features from the cells cultured under different light conditions (**Table 2** and **Figure 8**).

The decreased amount of total chlorophyll per gram cells under WL light conditions (**Figure 2**) is likely complemented by the dramatically increased amount of phycobiliprotein, compared with the higher chlorophyll content in the FR light grown cells. With the aid of phycobiliprotein to capture light under WL, OR or RL conditions, cells are unlikely to require as much chlorophyll as that which is needed under the FR light environment, beyond the phycobiliprotein absorption region. The mechanism of this pigment adaptation process remains undefined.

Pigment analysis of cell cultures grown under different light qualities demonstrate the increase in the PC index [phycobiliprotein to Chl *a* ratio (*A*_625-780 nm_/*A*_678-780 nm_)] for the cells grown under OR, RL or WL light, and the decrease of Chl *f* composition in total chlorophyll relative to FR light grown cells (**Figure 6A** insert). These results strongly suggest that *H. hongdechloris *acclimates to the shifted accessible light environment by varying the pigment composition. The experiments using a mixture of OR light/10 μE and FR light/10 μE light were also performed in order to investigate the effect of expanding the photosynthetic active spectral region from 600 to 750 nm. In this OR+FR light condition, *H. hongdechloris* retain both Chl *f* and phycobiliproteins (data not shown). However, the PC index decreased to around 0.5 ± 0.03 compared with that of cell grown under OR light/20 μE (0.62 ± 0.04) and Chl *f*/Chl *a* index decreased to about 0.20 ± 0.02 compared that of cell grown under FR light/20 μE (0.29 ± 0.03). Surprisingly, the coexistence of phycobiliproteins and Chl *f* does not appear to benefit *H. hongdechloris* growth with an even slower *k* of 0.11 ± 0.01 day^-1^compared to the *k* of 0.13 ± 0.01 day^-1^ when using OR light/20 μE or the *k* of 0.17 ± 0.01 day^-1 ^using FR light/20 μE light. The results suggest that the main light harvesting strategies of *H. hongdechloris *grown under FR light compared with <700 nm light are different and not complementary corresponding to one another. Although the light-harvesting strategy of *H. hongdechloris* is currently undefined, our data presented here indicates that *H. hongdechloris* uses phycobiliproteins as its major antenna component when grown under light sources <700 nm, but uses Chl *f* to access light beyond 700 nm (i.e*.*, grown under FR light). The relatively stable content of Chl *a* in *H. hongdechloris* under different light conditions strongly suggests that Chl *a* is the main photopigment in the photosystem reaction centers, although further experiments are required to confirm this hypothesis.

Previous results show that Chl *f* is a FR light induced chlorophyll (Akutsu et al., 2011; Chen et al., 2012) and phycobiliprotein contents increased dramatically in cell grown under WL, RL or OR light, which indicates photoacclimation between phycobiliproteins and chlorophyll-binding light-harvesting protein complexes (Gloag et al., 2007; Bibby et al., 2009). Both results were supported by confocal fluorescence results. **Figure 8A** illustrates that strong >692 nm fluorescence mainly came from Chl *f*, which was only detected in FR light-grown cells (Li et al., 2013). The much weaker but still visible >692 nm fluorescence observed in cells grown under WL, OR or RL light is most likely due to the presence of red-shifted Chl *a* in photosystems (Chen and Scheer, 2013). Fluorescence between 600 and 680 nm was observed strongly and uniformly around the periphery of cells grown under WL, RL or OR light, whereas in FR light-grown cells, this fluorescence was much weaker and concentrated at the septa (**Figure 9B**). Taken together with the pigment analyses (**Figure 6A**), the phycobiliprotein remanets in FR light grown cells seem predominantly localized at the septa of the cells or distributed between the plasma membrane and Th (**Figure 9B**). However, we cannot distinguish the different locations and correlation between phycobiliproteins and chlorophylls due to the resolution limits associated with confocal microscopy.

*Halomicronema hongdechloris* was isolated from a inner layer sediment of stromatolites which is a light filtered environment. Approximately 97% of light is attenuated through a 3 mm thick microbial mat (Brock, 1976; Al-Najjar et al., 2010). Therefore the light intensity inside stromatolites is expected to be extremely low, especially in the visible light spectral region. The photosynthetic microbial communities thriving in these unique niches have developed a capacity to flourish in such extreme light environment, which is consistent with the fact that *H. hongdechloris* prefers a lower light intensity and grows well under FR light.

**Figure 4A** illustrates that there were no significant reductions in growth rate when cells were grown at 27°C or 39°C compared with the optimal temperature 32°C (*F*(2, 21) = 1.07, *p* = 0.3617 > 0.05). However, these cells had approximately a week delay to reach the exponential phase when the temperature was decreased to 27°C (data not shown). In contrast, the cells grown at 39°C showed the similar growth profile as the cells at 32°C, but the growth rate was dropped dramatically after 10 days, without an obvious stationary phase (data not shown). One explanation for this observation is the reduced CO_2_ solubility in aqueous solution at higher temperature. The solubility of CO_2_ in water is about 1.45 g/L at 25°C; however, this decreases by more than 30% when the temperature is increased to 40°C (Carroll and Alan, 1992). This reduction in dissolved CO_2_ may inhibit photosynthetic efficiency. The culture plates have limited volumes and the surface area (2 ml testing culture in 3.6 ml vial), which may result in a limited gas exchange rate leading to a decreased CO_2_ concentration in the culture medium at higher temperatures.

*Halomicronema hongdechloris* grows well in 1 × seawater, but also tolerates higher salinity upto 4 × seawater. The initial cells are cultured under 1 × seawater control condition, therefore, the cells inoculated into 4 × seawater may face an osmotic shock. A long lag phase (~2 weeks) was observed, but the cells started to grow after 16 days (data not shown). This phenomenon is also observed in the cells grown in 3 × seawater. The longer lag phase may represent the periods when cells prepare themselves for adapting such hypersaline conditions, albeit only after more than two weeks exposed to the high salt. In other words, *H. hongdechloris* demonstrates halotolerant characteristics, which is consistent with the feature observed in the *Halomicronema* strain TEFP, a moderately halophilic cyanobacterium growing within the salinity range of 40–132‰ (Abed et al., 2002). Unsurprisingly, *Halomicronema* strain TEFP is the closest identified relative of *H. hongdechloris* based on 16S rRNA classification. Previous studies reported that the ecological niche where *H. hongdechloris *was isolated (Hamelin Pool, Shark Bay, Western Australia) is hypersaline (Bauld, 1984; Papineau et al., 2005), owing to the restricted exchange of water between the open ocean and the shallow bay and a high net evaporation rate witnessed in the water at low tide during hot season. The reports also showed that several halophilic Archaea have been isolated from Hamelin Pool (Papineau et al., 2005; Goh et al., 2006). These evidences suggest that the microbial communities at Hamelin Pool must be able to adapt to the hypersaline coastal environment, which might explain the high salt tolerance observed in *H. hongdechloris*.

In this study, we focused on the requirements of *H. hongdechloris* for inorganic nitrogen and phosphorus. *H. hongdechloris* biomass reached a maximum of 35.7 mg cell wet weight per ml when the medium was supplemented with fivefold of both nitrogen and phosphorus (**Figure 5**). Diverse responses to differential nitrogen versus phosphorus concentration were observed. While the negative relationship between *H. hongdechloris* growth rate and the high N:P ratio of 780 or the low N:P ratio of 7.8 indicated the importance of correlation between nitrogen and phosphorus. Further investigation on the correlation between growth rate of *H. hongdechloris* and the additional organic form nutrients (carbon and nitrogen) is required.

## CONCLUSION

In this study, we optimized the growth conditions for *H. hongdechloris*, both in terms of growth kinetics and Chl *f *content. We suggest the best culture conditions for *H. hongdechloris*, with a growth rate of 0.22 ± 0.02 day^-1^, are: FR light with an intensity of 20 μE, at 32°C, pH 8.0, salinity of 33‰ in modified K + ES seawater medium with increased nitrogen and phosphorus concentrations of 11.75 mM and 0.15 mM, respectively. *H. hongdechloris* cells are halotolerant, and do not grow in fresh water medium (salinity of 0‰). Sodium nitrate is the most favorable nitrogen sources among the tested inorganic nitrogen compounds. The greatest yield of Chl *f* is ~10% of the total chlorophyll under these assigned optimal culture conditions. The cells grown under WL, OR and RL light showed a very similar content of phycobiliprotein with a PC index of ~0.6. Fluorescence confocal microscopy reveals an unusual distinction in the distribution pattern of phycobiliproteins in Chl *f*-containing cells compared with those cells that do not have detectable Chl *f*. This result is supported by transmission electron microscopy analysis which shows a reduction in the number of phycobilisome-like structures present in a transverse section of Chl *f-*containing cells compared with cells that do not have Chl *f*. Different active O_2_ evolution was observed between Chl *f* containing FR light-grown cells and WL light-grown cells, when illuminated by FR light, which confirms the spectral expansion of oxygenic photosynthesis afforded by the presence of Chl *f *in *H. hongdechloris*.

## Conflict of Interest Statement

The authors declare that the research was conducted in the absence of any commercial or financial relationships that could be construed as a potential conflict of interest.

## Abbreviation

Car: carotenoid
Chl: chlorophyll
Cm: cytoplasmic membrane
FR: far-red light
fs: fibril sheath
*H. hongdechloris*: *Halomicronema hongdechloris*
HPLC: high performance liquid chromatography
*k*: growth rate
LEDs: light emitting diodes
N: nitrogen
OR: orange-red light
P: phosphorus
PC index: the relative amount of phycobiliprotein to Chl *a *ratio
Pg: peptidoglycan layer
PS: photosystem
*R*^2^: the coefficient of determination
RL: red light
*t*: doubling time
Th: thylakoid membranes
WL: cool white fluorescent light or white light
